# Heatstroke Is Associated with an Increased Risk of Chronic Headache: A Retrospective Cohort Study

**DOI:** 10.3390/brainsci15091011

**Published:** 2025-09-19

**Authors:** Karel Kostev, Ira Rodemer, Marcel Konrad, Jens Bohlken

**Affiliations:** 1 Epidemiology, IQVIA, Unterschweinstiege 2–14, 60549 Frankfurt am Main, Germany; ira.rodemer@iqvia.com; 2 University Hospital, Philipps University of Marburg, 35043 Marburg, Germany; 3 Health & Social, FOM University of Applied Sciences for Economics and Management, 60486 Frankfurt am Main, Germany; 4 Faculty of Medicine, Institute of Social Medicine, Occupational Health and Public Health, University of Leipzig, 04109 Leipzig, Germany

**Keywords:** migraine, heatstroke, cohort study, Germany

## Abstract

Background: Heatstroke is the most severe form of heat-related illness. It is characterized by an elevated core body temperature and central nervous system dysfunction. The aim of this study was to investigate the association between heatstroke and subsequent migraine development. Methods: This retrospective cohort study used data from the Disease Analyzer database (IQVIA) and included individuals diagnosed with heatstroke, as well as propensity score-matched individuals without heatstroke. Data about these individuals was recorded in 1216 general practices in Germany between January 2005 and December 2023. Five-year cumulative migraine incidence was assessed using Kaplan–Meiercurves, and univariable Cox regression analysis was performed to evaluate the association between heatstroke and migraine. Results: The study included 5794 individuals with heatstroke and 28,970 matched controls without heatstroke (median age: 30 years, 31–32% female). Most heatstroke cases were documented in June (32–34%), followed by July (30%), August (15–17%), and May (13%). Within five years of follow-up, 8.8% of patients with heatstroke and 4.0% of controls were diagnosed with migraine. The regression analysis revealed that heatstroke was significantly associated with an increased risk of migraine in the total population (HR: 2.26; 95% CI: 2.00–2.57), as well as in women (HR: 2.33; 95% CI: 1.96–2.79) and men (HR: 2.26; 95% CI: 1.89–2.70). Conclusion: This study highlights an important, yet previously underrecognized, association between heatstroke and an increased risk of migraine. As global temperatures continue to rise, public health strategies should focus not only on the acute prevention and management of heat-related illnesses, but also on their potential long-term neurological consequences.

## 1. Introduction

Heatstroke, the most severe form of heat-related illness, is characterized by an elevated core body temperature and central nervous system dysfunction [1]. As climate change increases the frequency and intensity of heat waves, the global public health burden of heatstroke continues to rise [2,3]. Traditionally regarded as an acute medical emergency, heatstroke is primarily associated with damage to the cardiovascular [4], neurological [5], renal [6] and hepatic systems [7], as well as increased mortality [1]. However, its potential long-term neurological consequences, particularly in relation to primary headache disorders, remain poorly understood.

Among these disorders, migraine, one of the most prevalent recurrent headache disorders worldwide [8,9], substantially reduces quality of life and productivity [10,11].

Increasing epidemiological and clinical evidence suggests that environmental stressors, particularly weather extremes, may not only serve as triggers but also as long-term contributors to headache pathophysiology [12,13].

The link between acute environmental exposures—such as high ambient temperature, dehydration, or barometric pressure changes—and headache exacerbation is well established. By contrast, the possibility that a single severe thermal insult, such as heatstroke, may initiate a persistent vulnerability to chronic headache disorders remains largely unexplored. This absence of long-term, population-based evidence represents an important research gap. Given that heatstroke can cause injury to the central nervous system—including disruption of the blood–brain barrier, cerebral edema, and inflammation—it is biologically plausible that it could lead to persistent neurological sequelae, including headache disorders [14]. However, population-based data supporting this association are scarce.

Severe hyperthermia can transiently compromise the blood–brain barrier (BBB) and blood–CSF interfaces, inducing vasogenic/cytotoxic edema and neuroinflammation—changes repeatedly described after heatstroke and proposed to underlie lasting neurological sequelae [14]. Recent experimental work further supports a temperature-driven BBB insult: in heat-stressed mice, endothelial-stabilizing treatment mitigated BBB leakage and cognitive deficits, consistent with BBB disruption as an upstream event after thermal injury [15]. BBB perturbation can amplify inflammatory signaling within pain-relevant circuits and lower the threshold for central sensitization.

In parallel, hyperthermia can engage heat-sensitive transient receptor potential (TRP) channels (e.g., TRPV1) expressed on trigeminal afferents. Activation of these channels promotes release of calcitonin gene-related peptide (CGRP) from meningeal/trigeminal terminals, facilitating vasodilation and neurogenic inflammation within the trigeminovascular system [16]. CGRP, in turn, is a well-established effector in migraine biology: human and translational evidence shows CGRP elevations during attacks and robust anti-migraine efficacy when the CGRP pathway is blocked, providing biological plausibility that heatstroke-level hyperthermia could precipitate migraine in susceptible individuals [17,18].

This study aims to examine whether individuals in Germany who experience heatstroke are at an increased long-term risk of being diagnosed with migraines.

## 2. Methods

### 2.1. Database

This retrospective cohort study used the Disease Analyzer database (IQVIA), which contains anonymized information on prescriptions, diagnoses, and basic demographic and medical characteristics. This information is obtained directly from the computer systems used in the practices of general practitioners and specialists [19]. The database covers approximately 3000 office-based physicians in Germany and has been validated as representative of general and specialized practices nationwide [19].

### 2.2. Study Population

**Figure 1:**
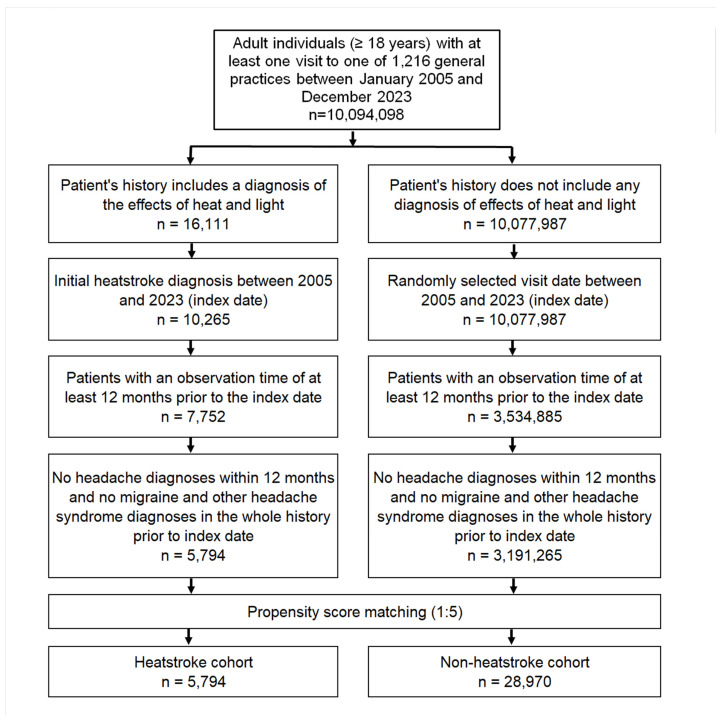
Selection of study patients.

This study included adult patients (≥18 years) who received an initial diagnosis of heat stroke (ICD-10: T67.0) in one of 1216 general practices in Germany between January 2005 and December 2023 (index date; Figure 1). To ensure adequate access to co-diagnoses, only patients with at least 12 months of documented medical history prior to the index date were included. Patients with a diagnosis of migraine (ICD-10: G43) or other headache syndromes (ICD-10: G44) at any time prior to or on the index date, as well as those with a diagnosis of headache (ICD-10: R51, G44) within the 12 months preceding the index date, were excluded.

After applying similar inclusion criteria, individuals whose medical history did not include a diagnosis of the effects of heat and light (ICD-10: T67) were matched with heatstroke patients using nearest neighbor propensity score matching (5:1) based on age, sex, index year, month, and co-diagnoses possibly related to headaches. These co-diagnoses included diabetes mellitus (ICD-10: E10–E14), hypertension (ICD-10: I10), alcohol addiction (ICD-10: F10), depression (ICD-10: F32, F33), reaction to severe stress and adjustment disorders (ICD-10: F43), and sleep disorders (ICD-10: G47, F51) documented within 12 months prior to or on the index date. For the non-heatstroke cohort, the index date was that of a randomly selected visit between January 2005 and December 2023 (Figure 1). The covariate balance between the cohorts was assessed using the standardized mean difference (SMD). Values <0.1 indicate an acceptable covariate balance.

### 2.3. Study Outcomes

The outcome of the study was an initial migraine diagnosis (ICD-10: G43) within 5 years following the index date as a function of heatstroke.

### 2.4. Statistical Analyses

The five-year cumulative incidence of migraine was studied using Kaplan–Meier curves. To evaluate the association between heatstroke and subsequent migraine diagnosis, a univariable Cox regression analysis was performed. Multivariable regression was not performed because propensity score matching based on demographic and clinical variables resulted in well-balanced cohorts (all standardized mean differences <0.1), and therefore additional adjustment was not necessary. The analyses were stratified by age group (≤22, 23–30, 31–45, >45) based on the 25% quantile, median, and 75% quantile of the age distribution, as well as by sex. Results from the Cox regression model are reported as hazard ratios (HRs) with 95% confidence intervals (CIs). A *p*-value of <0.05 was considered statistically significant. The analyses were performed using SAS version 9.4 (SAS Institute, Cary, NC, USA).

## 3. Results

### 3.1. Basic Characteristics of the Study Sample

The study included 5794 individuals with a documented heatstroke diagnosis and 28,970 matched controls without heatstroke. The baseline characteristics of the study population are displayed in Table 1. The median age across the cohorts was 30 years, with women comprising approximately 31–32% of each group. Propensity score matching ensured good balance of baseline characteristics between cohorts. Most heatstroke diagnoses were documented in June (32–34%), followed by July (30%), August (15–17%), and May (13%).

### 3.2. Cumulative Incidence of Migraine

**Figure 2:**
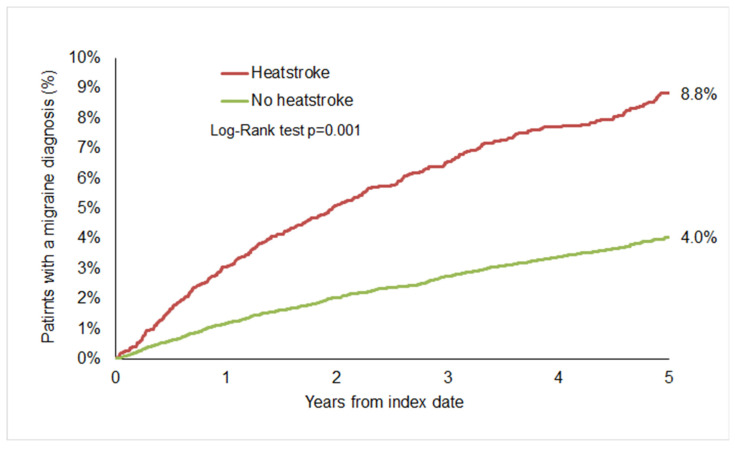
Cumulative incidence of migraine in patients with and without heatstroke.

Over a follow-up period of up to five years, migraine was diagnosed in 8.8% of patients in the heatstroke group, compared to 4.0% in the control group (Figure 2). Among those diagnosed with migraine, 82.8% of the heatstroke cohort and 83.2% of the non-heatstroke cohort were classified under ICD-10: G43.9 (migraine, unspecified). Migraine without aura was documented in 6.8% of the heatstroke cohort and 5.5% of the non-heatstroke cohort, while migraine with aura was recorded in 7.3% and 7.5%, respectively. Other migraine subtypes were rarely reported.

### 3.3. Association Between Heatstroke and Subsequent Migraine

Regression analysis revealed a significant association between heatstroke and an elevated risk of developing migraine (HR: 2.26; 95% CI: 2.00–2.57). This association was consistent across sexes, with HRs of 2.33 (95% CI: 1.96–2.79) in women and 2.26 (95% CI: 1.89–2.70) in men. The association remained significant across all age groups, ranging from an HR of 1.86 (95% CI: 1.40–2.47) in individuals aged 31–45 years to an HR of 2.94 (95% CI: 2.01–4.29) in those over 45 years old (Table 2).

In sensitivity analyses restricted to more specific migraine diagnoses, heatstroke remained significantly associated with migraine with aura (G43.0: HR 3.03; 95% CI: 1.85–4.96) and migraine without aura (G43.1: HR 2.32; 95% CI: 1.48–3.64).

## 4. Discussion

This large retrospective cohort study provides the first population-based evidence that heatstroke is associated with an increased long-term risk of developing migraine. While previous case reports and mechanistic studies have suggested a possible link, our findings extend this knowledge by demonstrating the association at scale in routine clinical practice.

This association was robust across both sexes and most age groups, highlighting the potential for lasting neurological consequences of heatstroke. Notably, a substantial proportion of the study population was young. While detailed contextual data were unavailable, it is plausible that younger people are more likely to engage in strenuous outdoor activities, such as sports, exercise, or physically demanding work during the summer months. These activities can elevate core body temperature and lead to dehydration. Contributing factors may include inadequate hydration, consumption of energy drinks or alcohol, and limited awareness of the early signs of heat exhaustion [20].

Beyond clinical heatstroke, prior epidemiological studies indicate that ambient heat itself may independently increase migraine risk. For example, population-based analyses have linked rising outdoor temperatures and heat waves to higher rates of headache-related emergency visits and migraine onset [21,22]. Our dataset does not allow separation of migraine risk attributable to clinical heatstroke from that attributable to ambient thermal exposures alone. Nevertheless, acknowledging both pathways provides a broader context: heatstroke may represent an extreme end of a continuum in which environmental heat stress contributes to migraine pathogenesis.

The observed association between heatstroke and migraine (HR: 2.26) is notable. Migraines are complex neurological disorders involving neurovascular and inflammatory processes, and an episode of heatstroke could potentially exacerbate or initiate these processes.

Heatstroke typically leads to hyperthermia, which can cause lasting or even permanent neurological effects. Heatstroke and hyperthermia can activate harmful processes in the brain, including excitotoxicity, mitochondrial dysfunction, local inflammation, and cytokine release [23].

Notably, many of these mechanisms have been implicated in migraine pathogenesis. First, migraines are associated with cortical spreading depression (CSD)—a wave of neuronal and glial depolarization followed by a period of suppressed brain activity. CSD is linked to excitotoxicity, particularly involving glutamate pathways [24]. Second, impaired energy metabolism, especially involving mitochondrial dysfunction, is thought to be a factor in certain migraine subtypes, particularly familial hemiplegic migraine and migraine with aura [25]. Third, neurogenic inflammation involving the trigeminovascular system leads to the release of pro-inflammatory neuropeptides and cytokines, along with vasodilation [26,27]. Finally, changes in cerebral blood flow are frequently observed, particularly in cases of migraine with aura [28].

Several limitations of the study should be acknowledged. First, relying on routinely collected outpatient data introduces the potential for misclassification bias. Since heatstroke is a severe, acute condition, many diagnoses may reflect encounters after hospitalization rather than the acute phase itself. Furthermore, as heatstroke is associated with mortality, many patients captured in outpatient records may have experienced milder cases. Information on the severity of heatstroke and hospitalization status was unavailable. Both could influence long-term outcomes. Contextual details, such as the setting of heatstroke onset (e.g., occupational exposure vs. recreational activity), were also unavailable.

While diagnoses in the Disease Analyzer database are recorded by physicians, coding errors or inconsistencies in migraine documentation may exist. This is particularly relevant for migraine diagnoses, where a substantial proportion of cases were coded as “migraine, unspecified” (ICD-10: G43.9), representing a further limitation. Furthermore, while recent clinical coding allows differentiation between unspecified and severe heatstroke (e.g., ICD-10 sub-code T67.0A), our database only stores diagnoses up to one digit after the decimal point. This prevented us from stratifying the cohort by heatstroke severity. Consequently, it remains unclear whether migraine risk differs between milder and more severe heatstroke cases, and future datasets with more granular coding could help address this question. Additionally, we were unable to account for several unmeasured confounders, including family history of headaches, socioeconomic status, occupation, smoking status, and detailed environmental exposures. It is plausible that factors associated with both heat exposure risk and headache susceptibility, such as outdoor labor or low socioeconomic status, were unevenly distributed despite matching. Although propensity score matching ensured balance in measured covariates, the possibility of residual confounding from unmeasured factors—such as socioeconomic status, occupational exposures, and family history of headache—cannot be excluded. Moreover, it remains unclear whether post-heatstroke headaches represent new-onset primary headache disorders or secondary headaches resulting from neurological sequelae. Although individuals with prior migraine or other headache syndrome diagnoses were excluded, the possibility of subclinical or undiagnosed headaches prior to heatstroke cannot be ruled out. It is also possible that at least a subset of the post-heatstroke headache diagnoses coded as migraine may, in fact, represent secondary headache disorders due to structural or inflammatory sequelae of heatstroke rather than new-onset primary migraine. The predominance of unspecified migraine codes (G43.9) in our dataset makes this distinction difficult. However, our sensitivity analyses restricted to more specific ICD-10 codes (migraine with aura, G43.0, and without aura, G43.1) confirmed robust associations, suggesting that misclassification alone is unlikely to explain our findings.

Accordingly, while the observed hazard ratio of ~2.3 indicates a robust association, this effect size should be interpreted with caution. Coding inaccuracies in migraine diagnoses and residual confounding from unmeasured factors (e.g., socioeconomic status, occupation, family history) could have inflated or attenuated the true magnitude of risk.

Despite these limitations, the study has several notable strengths. The large sample size, representative dataset, and long follow-up period enhance the robustness and generalizability of the findings. The consistent association across multiple subgroups and sensitivity analyses further supports the reliability of the results.

In conclusion, this study provides population-based evidence of an association between heatstroke and an increased long-term risk of migraine. While causality cannot be established, the findings highlight the need for heightened clinical awareness of potential neurological sequelae following heatstroke. Individuals with a history of heatstroke may benefit from monitoring for the emergence of headache disorders. As global temperatures continue to rise, public health strategies should consider not only the acute prevention and management of heat-related illnesses, but also their possible long-term neurological consequences.

## Figures and Tables

**Table 1:** Baseline characteristics of the study sample (after 1:5 propensity score matching).

Variable	Proportion Among Patients with Heatstroke (*n*, %) *n* = 5794	Proportion Among Patients Without Heatstroke (*n*, %) *n* = 28,970	SMD
Age (Median, IQR)	30 (22–45)	30 (22–44)	0.003
Age 18–22 years	1501 (25.9)	7354 (25.4)	
Age 23–30 years	1460 (25.2)	7385 (25.5)	
Age 31–45 years	1466 (25.3)	7485 (25.8)	
Age >45 years	1367 (23.6)	6746 (23.3)	
Female	1818 (31.4)	9198 (31.7)	−0.004
Male	3976 (68.6)	19,772 (68.3)	
Year of study inclusion			0.001
2005–2008	262 (4.5)	1473 (5.1)	
2009–2012	507 (8.8)	2487 (8.6)	
2013–2016	1020 (17.6)	4995 (17.2)	
2017–2020	1987 (34.3)	9651 (33.3)	
2021–2023	2018 (34.8)	10,364 (35.8)	
Month of diagnosis			
May	756 (13.0)	3658 (12.6)	
June	1994 (34.4)	9394 (32.4)	
July	1742 (30.1)	8612 (29.7)	−0.017
August	852 (14.7)	4972 (17.2)	
Other months	450 (7.8)	2334 (8.1)	
Diabetes mellitus	285 (4.9)	1302 (4.5)	−0.004
Hypertension	1030 (17.8)	4912 (17.0)	−0.008
Sleep disorders	609 (10.5)	2888 (10.0)	−0.005
Depression	889 (15.3)	4277 (14.8)	−0.006
Reaction to severe stress	896 (15.5)	4289 (14.8)	−0.007
Alcohol addiction	184 (3.1)	768 (2.6)	−0.005

Proportions of patients presented as *n*, %, unless otherwise indicated. IQR: Interquartile Range. SMD: Standardized mean difference.

**Table 2:** Association between heatstroke and subsequent migraine diagnoses in patients followed in general practices in Germany (univariable Cox regression models).

Patient Subgroup	HR (95% CI)	*p* Value
Total	2.26 (2.00–2.57)	<0.001
Age 18–22 years	2.24 (1.83–2.75)	<0.001
Age 23–30 years	2.29 (1.82–2.87)	<0.001
Age 31–45 years	1.86 (1.40–2.47)	<0.001
Age >45 years	2.94 (2.01–4.29)	<0.001
Female	2.33 (1.96–2.79)	<0.001
Male	2.26 (1.89–2.70)	<0.001

## Data Availability

The data used in this study were obtained from IQVIA and are available upon reasonable request, subject to permission from IQVIA and applicable data protection regulations. The data are not publicly available due to privacy restrictions.
